# Incidence of stroke in patients with HIV infection: A population-based study in Taiwan

**DOI:** 10.1371/journal.pone.0217147

**Published:** 2019-05-22

**Authors:** Hui-Lin Lin, Chih-Hsin Muo, Cheng-Yu Lin, Hsuan-Ju Chen, Pei-Chun Chen

**Affiliations:** 1 PHD Program for Aging, China Medical University, Taichung, Taiwan; 2 Department of Physical Medicine and Rehabilitation, Lin Shin Hospital, Taichung, Taiwan; 3 Department of Public Health, China Medical University, Taichung, Taiwan; 4 Management Office for Health Data, China Medical University Hospital, Taichung, Taiwan; Azienda Ospedaliera Universitaria di Perugia, ITALY

## Abstract

**Background:**

Few studies have evaluated whether people infected with human immunodeficiency virus (HIV) are at an increased risk of stroke in an Asian population. We investigated the association between HIV infection and the risk of developing stroke by age, calendar year of HIV diagnosis, and follow-up duration in Taiwan.

**Methods:**

Using the claims data of a universal health insurance program, we identified 5,961 patients with HIV and 23,844 matched non-HIV subjects without previous stroke from 1998 to 2005 and followed them up until the end of 2011 to measure the incidence of stroke. Cox proportional hazards models adjusted for potential confounders were used to estimate hazard ratios (HR) and 95% confidence intervals (CI), with the non-HIV group as reference.

**Results:**

During a median follow-up of 8 years, the incidence rates for total, ischemic, and hemorrhagic stroke per 1000 person-years were 2.12, 1.22, and 0.60, respectively, in patients with HIV infection, and 1.98, 1.14, and 0.54, respectively, in the comparison group. HIV infection was associated with an elevated risk of developing total stroke (adjusted HR [95% CI], 1.57 [1.15–2.14]) and ischemic stroke (1.91 [1.25–2.91]) in patients aged less than 45 years, but no association was observed in other age groups (*P* for interaction with age, p = 0.048 and 0.024, respectively). Patients diagnosed with HIV infection in 1998–1999 had a greater HR for total stroke and ischemic stroke than those diagnosed in 2000–2002 and 2003–2005 (*P* for interaction, for total stroke p = 0.034, for ischemic stroke p = 0.056). The HRs did not differ by follow-up duration.

**Conclusions:**

HIV infection among a young age group is associated with increased risk of developing overall and ischemic stroke. The findings highlight the importance of screening and correcting risk factors for young stroke prevention immediately and aggressively.

## Introduction

Human immunodeficiency virus (HIV) infection largely affects sexually active young adults. With the development of the combination antiretroviral therapy (cART) regimen, used for people living with HIV/AIDS (PLWHA) since 1996, HIV replication was effectively inhibited, leading to a reduction of the risk of developing an AIDS-defining complication and prolonging the lifespan of patients with HIV [1]. However, the health of effectively treated patients with HIV is not fully restored and has led to a higher prevalence of the disease than in those without HIV infection [2].

Stroke is a significant cause of death and disability worldwide [3]. Acute or chronic infection is well recognized to be a contributing factor in strokes and can influence the outcome of strokes [4]. Several studies have reported the association between HIV infection and the risk of stroke [1, 5–17]. However, most of these studies have been confined to Western populations [1, 5–12, 15–17]. Little is known regarding the association between HIV infection and the risk of stroke in Asian people [6, 13, 14]. Multiple factors, including age, sex, family history, the effect of HIV itself, the effect of antiretroviral therapy, traditional cardiovascular risk factors etc., influence the risk of stroke in HIV-infected individuals. Genetics in different populations also play a role. Epidemiology data have been revealed to be different in Western and Asian countries due to the influence of population-specific phenotypic effects and gene susceptibility on the progression of HIV infection [18, 19]. Black people have shown more prominent cerebrovascular endothelial dysfunction leading to an elevated risk of stroke compared with other race/ethnic groups after adjusting for several traditional vascular risk factors in an ART-treated PLWHA group [20]. A recent multiethnic study revealed that the incidence rate of stroke was greater in non-Hispanic Black people than in other ethnic groups in persons living with HIV infection, but only 2% of the study subjects (n = 116) were Asian/Pacific Islanders [15]. Furthermore, studies of the US population have shown a greater risk of stroke in women and in younger people [8, 12, 15, 16], but limited data is available for other ethnic groups. A recent population-based cohort study in Taiwan reported that patients that are HIV-positive had an increased risk of stroke, as compared with individuals who are HIV-negative [14]. The incidence rate of stroke among individuals that are HIV-infected in that study [14] was lower than that in previous reports in Western countries [5, 7–9, 11, 12]. However, data by types of stroke and whether the HIV-related risk of stroke differs by age and sex was not reported in that study [14]. Furthermore, little is known regarding the risk of stroke in association with HIV infection by calendar years of diagnosis [21] and the duration of follow-up. Using a national database from Taiwan, we explored the risk of overall and different types of stroke in patients with HIV infection by age, sex, calendar year of diagnosis, and follow-up years.

## Methods

### Study design and data source

We conducted a retrospective cohort study using claims data of a universal health insurance program in Taiwan [22]. The National Health Insurance (NHI) program is a mandatory single-payer national health insurance program providing comprehensive healthcare coverage to more than 99% of the 23 million people in Taiwan [23]. The NHI claims data is provided to scientists for research purposes, and all personal identification information was encrypted for the protection of patient privacy [6]. This study was approved by the Institutional Review Board of China Medical University & Hospital (CRREC-106-074).

### Identification of patients living with HIV infection and the comparison group

**Fig 1:**
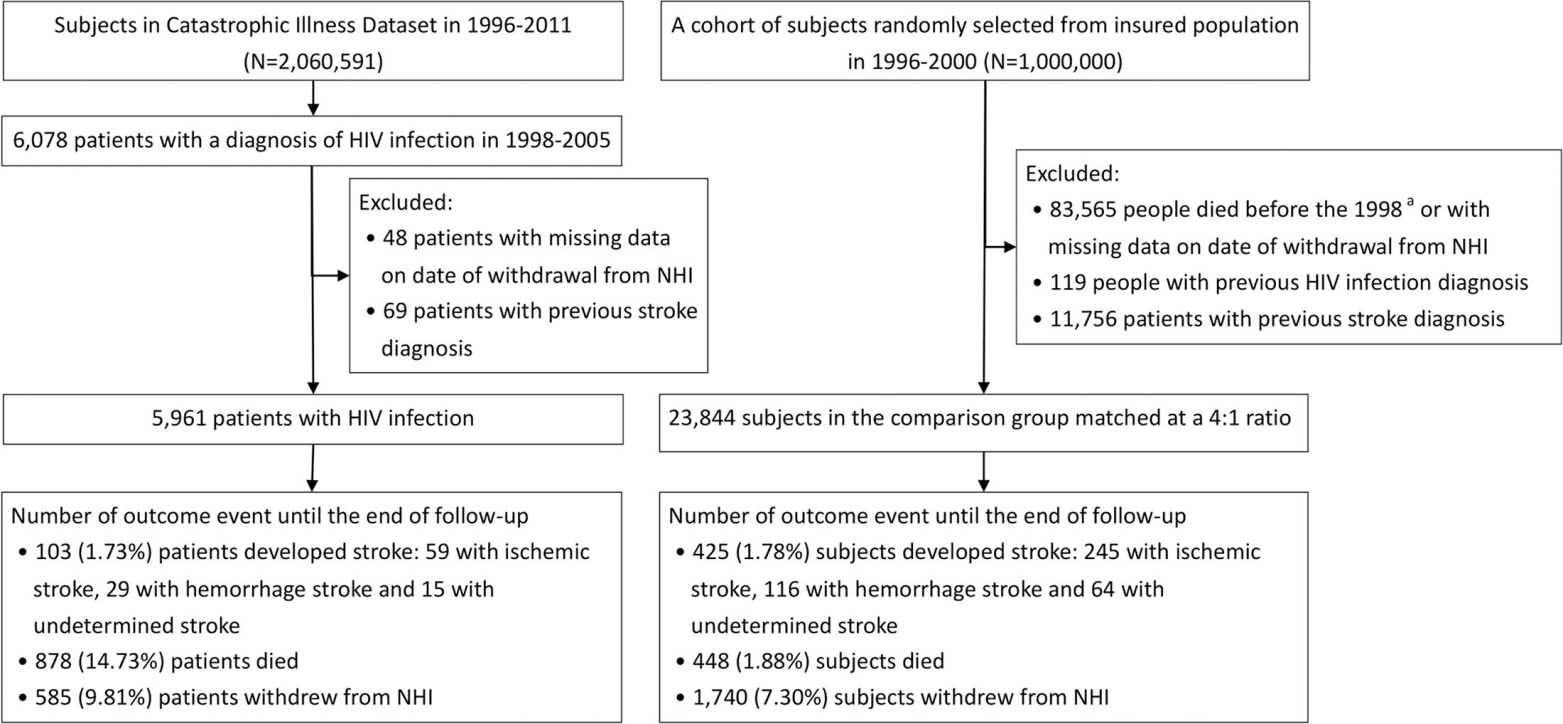
Flow chart of study subjects’ selection. ^a^Death was defined by using hospital discharged records and registry of catastrophic illness dataset. NHI indicates National Health Insurance Program; HIV, human immunodeficiency virus.

Fig 1. shows the flow chart for the subject selection process. We identified a cohort of patients diagnosed with HIV infection for the first time during the period of 1 January 1998 and 31 December 2005, according to the International Classification of Diseases, Ninth Revision, Clinical Modification (ICD-9-CM) codes 042 and V08 listed in the Registry for Catastrophic Illness Patient Database. Taiwan’s NHI defines 31 categories of catastrophic illness, including HIV infection. When patients are diagnosed and confirmed as having such definite catastrophic illnesses, they are qualified to apply for catastrophic illness certificates by their attending physician, and thus become exempt from copayments. To apply for the catastrophic illness certificates for HIV infection, patients’ infectious disease physicians must provide pertinent medical records and data for formal review, including clinical histories, the definite positive results of the HIV antibody and antigen combination assays in the laboratory tests, and the results of viral load and CD4 count examinations to validate the diagnoses. An expert committee composed of infectious disease physicians issues catastrophic illness certificates of HIV after a review of the applications. For each of the patients with HIV, the date on which he or she was registered for the catastrophic illness served as the index date. Individuals who had received stroke diagnoses before the index date were excluded from the data analysis.

Using a dataset containing NHI claims of one million subjects randomly selected from the insured population during 1996–2000 (the Longitudinal Health Insurance Database [24]), we randomly selected patients without an HIV diagnosis to form a comparison group. Subjects in the comparison group were frequency matched with those in the HIV infection group at a 4:1 ratio based on age (every 5 years), sex, and the calendar year of the HIV diagnosis and were randomly assigned index dates in the same year of the HIV diagnosis in the HIV infection group. Individuals previously diagnosed with stroke were excluded.

### Follow-up for stroke development

Subjects in the HIV infection group and the comparison group were observed for the occurrence of stroke, defined as a hospital discharge diagnosis with stroke (ICD-9-CM codes 430–438). We classified patients with stroke into three categories: hemorrhagic stroke (ICD-9-CM codes 430–432), ischemic stroke (ICD-9-CM codes 433, 434, and 435.9), and undetermined type of stroke (ICD-9-CM codes 435–438 exclude 435.9). The follow-up period started on the index date and ended at the earliest of the following dates: stroke occurrence, withdrawal from the NHI program, or 31 December 2011.

### Baseline comorbidities

Comorbidities including diabetes mellitus (ICD-9-CM codes 250), hypertension (ICD-9-CM codes 401–405), hyperlipidemia (ICD-9-CM codes 272), chronic kidney disease (ICD-9-CM codes 580–587), cancer (ICD-9-CM codes 140–208), coronary heart disease (ICD-9-CM codes 410–414), and atrial fibrillation (ICD-9-CM codes 427.31), which are known risk factors of stroke, were considered as potential confounders (S1 Table). All comorbidities were identified by the presence of diagnosis codes in at least two outpatient claims or one hospital inpatient claim within two years before the index date.

### Statistical analysis

We compared the baseline characteristics of patients with HIV and the comparison group using *t*-tests for continuous variables and chi-squared tests for categorical variables. For each group, the incidence density rates of stroke were calculated by using the number of patients with incident stroke events divided by total follow-up person-years. We used Cox proportional hazards models, which yielded hazard ratios (HR) and 95% confidence intervals (CI), to assess the risk of developing total, ischemic, and hemorrhagic stroke in association with HIV infection. The models were adjusted for age, sex, and the comorbidities to control for potential confounding factors. The Cox models were performed in patient subgroups stratified by age, year of HIV diagnosis, and follow-up period to observe whether the risks differ between the stratifications. Interaction effects of age and year of diagnosis with HIV were examined by using likelihood ratio tests comparing Cox models excluding and including the interaction terms. The proportional hazards assumption, i.e., whether the risk differs by follow-up time, was also tested by including interaction terms between the HIV infection and a function of follow-up time in the models. We used the SAS 9.4 software package (SAS Institute, Cary, NC USA) to perform all data management and analyses.

## Results

### Characteristics of subjects in the HIV-infected group and the comparison group

There were 6,078 patients with HIV infection identified from 1998 to 2005 (Fig 1). We excluded subjects with missing or invalid data on the date of withdrawal from the NHI program, and those with a previous diagnosis of stroke. The remaining 5,961 patients in the HIV infection group were included in the analysis. A total of 23,844 subjects without HIV infection who met the inclusion criteria were selected in the age-, sex-, and calendar year-matched comparison group.

The ratio of male to female subjects in the HIV-infection cohort was nearly 11.8 (crude number 5495 vs. 466; 92.2% vs. 7.8%) (Table 1). The mean (standard deviation, SD) age at HIV diagnosis was 34.2 (10.9) years for male patients and 36.4 (12.7) years for female patients. Of patients living with HIV infection, 61% had their first diagnosis at age 35 years or younger. The median follow-up time to occurrence of stroke was 8.1 years for the HIV group and 8.4 years for the comparison group (interquartile range, 4.2 years vs. 5.6 years). The mean (SD) age of diagnosis with stroke in the HIV infection cohort was younger than that in the comparison group (50.8 [15.3] years vs. 57.3 [14.3] years, p<0.0001). Relative to subjects in the comparison group, patients with HIV infection were more likely to have comorbid diabetes (3.1% vs. 2.2%), chronic kidney disease (1.6% vs. 0.7%), and cancer (1.0% vs. 0.4%).

**Table 1:** Characteristics of patients with HIV infection and subjects in the comparison group.

Variable	Patients with HIV (n = 5961)		Comparison group (n = 23844)		p-value
**Sex**^a^ **(n (%))**					N.A.
** Male subjects**	5495	92.2	21980	92.2	
** Female subjects**	466	7.8	1864	7.8	
**Age at HIV diagnosis, years (n (%))**^a^					N.A.
** 0–25**	1101	18.5	4404	18.5	
** 25–35**	2534	42.5	10136	42.5	
** 35–45**	1468	24.6	5872	24.6	
** 45–55**	491	8.2	1964	8.2	
** 55–65**	248	4.2	992	4.2	
** ≥65**	119	2.0	476	2.0	
** Median (mean ± SD)**^b^					
** All**	32.3 (34.3 ± 11.1)		32.4 (34.3 ± 11.3)		
** Male subjects**	32.2 (34.2 ± 10.9)		32.3 (34.2 ± 11.1)		
** Female subjects**	33.9 (36.4 ± 12.7)		33.9 (36.3 ± 12.9)		
**Comorbidities**^a^					
** Diabetes**	183	3.1	514	2.2	<0.0001
** Hypertension**	245	4.1	1060	4.5	0.26
** Hyperlipidemia**	137	2.3	626	2.6	0.15
** Chronic kidney disease**	97	1.6	170	0.7	<0.0001
** Cancer**	60	1.0	101	0.4	<0.0001
** Coronary heart disease**	99	1.7	339	1.4	0.17
** Atrial fibrillation**	9	0.2	20	0.1	0.14
**Duration of the follow-up, years (median, (interquartile range))**^c^					
** Male subjects**	8.1 (4.1)		8.4 (5.5)		<0.0001
** Female subjects**	7.6 (4.9)		8.4 (5.6)		<0.0001
** All**	8.1 (4.2)		8.4 (5.6)		<0.0001
**Age at diagnoses of stroke, years (median (mean ± SD))**^b^					
** Male subjects**	48.6 (50.3 ± 15.7)		56.8 (57.2 ± 14.5)		<0.0001
** Female subjects**	51.1 (54.3 ± 12.4)		61.0 (59.2 ± 11.3)		0.22
** All**	49.1 (50.8 ± 15.3)		58.1 (57.3 ± 14.3)		<0.0001

Abbreviations: HIV, human immunodeficiency virus; N.A., not applicable; SD, standard deviation.

^a^Chi-squared test.

^b^T-test.

^c^Wilcoxon Rank sum test.

### HIV infection and the risk of stroke by age and sex

Table 2 shows the incidence rates and HRs for stroke overall and in subgroups stratified by age and sex. During the follow-up period, the incidence rates for total, ischemic, and hemorrhagic stroke per 1000 person-years were 2.12, 1.22, and 0.60, respectively, in the patients with HIV infection. The corresponding rates in the comparison group were 1.98, 1.14, and 0.54. In the multivariable-adjusted models, overall we did not observe a statistically significant association between HIV infection and the risk of developing total stroke (adjusted HR [95% CI], 1.21 [0.98–1.51]), ischemic stroke (1.23 [0.93–1.64]), and hemorrhagic stroke (1.18 [0.78–1.78]). The adjusted HR for total stroke was greater among women than in men (adjusted HR [95% CI], 2.25 [1.15–4.41] and 1.15 [0.91–1.44], respectively), but the sex difference was not statistically significant (for the interaction with sex p = 0.085).

**Table 2:** Risk of stroke in relation to HIV infection by age and sex.

Age group, years	Total stroke^a^					Ischemic stroke					Hemorrhage stroke				
	HIV group		Comparison group		HR (95% CI)^b^	HIV group		Comparison group		HR (95% CI)^c^	HIV group		Comparison group		HR (95% CI)^c^
	No. event	Rate^b^	No. event	Rate^b^		No. event	Rate^b^	No. event	Rate^b^		No. event	Rate^b^	No. event	Rate^b^	
**All**															
**<45**	55	1.29	155	0.84	1.57 (1.15–2.14)	31	0.73	73	0.40	1.91 (1.25–2.91)	16	0.38	66	0.36	1.07 (0.62–1.84)
**45–65**	34	6.36	196	7.23	0.82 (0.57–1.19)	20	3.74	121	4.47	0.76 (0.47–1.23)	10	1.87	45	1.66	1.09 (0.54–2.18)
**>65**	14	23.57	74	20.84	1.08 (0.60–1.94)	8	13.47	51	14.36	0.91 (0.63–1.96)	3	5.05	5	1.41	3.07 (0.66–14.3)
**All**	103	2.12	425	1.98	1.21 (0.98–1.51)	59	1.22	245	1.14	1.23 (0.93–1.64)	29	0.60	116	0.54	1.18 (0.78–1.78)
**Male subjects**															
**<45**	49	1.23	146	0.85	1.47 (1.06–2.04)	27	0.68	67	0.99	1.80 (1.15–2.82)	15	0.38	64	0.37	1.02 (0.58–1.80)
**45–65**	28	6.09	179	7.71	0.77 (0.52–1.15)	16	3.48	110	4.74	0.70 (0.41–1.19)	8	1.74	41	1.77	1.00 (0.47–2.13)
**>65**	13	24.15	69	21.28	1.09 (0.59–2.00)	8	14.86	48	14.80	0.98 (0.45–2.11)	3	5.57	5	1.54	3.07 (0.66–14.3)
**All**	90	2.00	394	2.00	1.15 (0.91–1.44)	51	1.13	225	1.14	1.17 (0.86–1.58)	26	0.58	110	0.56	1.12 (0.73–1.72)
**Female subjects**															
**<45**	6	2.16	9	0.69	3.57 (1.24–10.3)	4	1.44	6	0.46	3.29 (0.93–11.7)	1	0.36	2	0.15	4.19 (0.30–58.2)
**45–65**	6	7.98	17	4.39	1.57 (0.56–4.40)	4	5.32	11	2.84	1.82 (0.54–6.10)	2	2.66	4	1.03	1.35 (0.13–13.8)
**>65**	1	17.97	5	16.19	1.79 (0.19–17.3)	0	0.00	3	9.71	NA	0	0.00	0	0.00	-
**All**	13	3.63	31	1.81	2.25 (1.15–4.41)	8	2.23	20	1.17	2.16 (0.93–5.01)	3	0.84	6	0.35	2.11 (0.41–10.8)

Abbreviations: CI, confidence interval; HIV, human immunodeficiency virus; HR, hazard ratio.

^a^Patients with undetermined stroke were excluded in the analysis by stroke subtypes (n = 79).

^b^Incidence rate per 1000 person-years.

^c^Adjusted for age, sex, and comorbidities including diabetes, hypertension, hyperlipidemia, chronic kidney disease, cancer, coronary heart disease and atrial fibrillation.

P values of test for interaction effect between age and HIV: all subjects, 0.048 for overall stroke, 0.024 for ischemic stroke and 0.386 for hemorrhage stroke; male patients, Interaction p 0.056 for overall stroke, 0.034 for ischemic stroke and 0.354 for hemorrhage stroke; female patients, I0.610 for overall stroke, 0.344 for ischemic stroke and 0.990 for hemorrhage stroke. P values of test for interaction effect between sex and HIV: 0.085 for overall stroke, 0.214 for ischemic stroke and 0.306 for hemorrhage stroke.

The age-stratified analyses showed that HIV infection was associated with an increased risk of total stroke and ischemic stroke in subjects <45 years of age (adjusted HR [95% CI], 1.57 [1.15–2.14] and 1.91 [1.25–2.91], respectively), but not in other age stratifications (for interaction with total stroke p = 0.048, for interaction with ischemic stroke p = 0.024). Similarly, the HRs differed significantly by age among men. Increased risk was found in men <45 years of age but not in older subjects (for interaction with total stroke p = 0.056, for interaction with ischemic stroke p = 0.034). Elevated HRs in young patients were also noted among women, but the interaction effect with age was not statistically significant. However, we did not observe associations between HIV infection and the risk of developing hemorrhagic stroke.

### HIV infection and the risk of stroke by year of diagnosis

Table 3 shows the association between HIV infection and the risk of developing stroke in analyses stratified by year of diagnosis of HIV infection. The adjusted HR for total stroke was greater in patients diagnosed with HIV infection in 1998–1999 (HR = 1.51, 95% CI = 1.08–2.10) than those in 2000–2002 (HR = 0.75, 95% CI = 0.48–1.18) and 2003–2005 (HR = 1.44, 95% CI = 0.98–2.11) (p for interaction, p = 0.034) (Table 3). Similar results were observed for ischemic stroke but not for hemorrhagic stroke. Patients diagnosed with HIV infection in 1998–1999 had an increased risk of ischemic stroke (HR = 1.73, 95% CI = 1.13–2.65), but no significant association was observed in those diagnosed in 2000–2002 (HR = 0.68, 95% CI = 0.39–1.28) and 2003–2005 (HR = 1.33, 95% CI = 0.80–2.20) (p for interaction, p = 0.056) (Table 3).

**Table 3:** Risk of stroke in patients with HIV infection according to calendar year of diagnosis.

Year of diagnosis of HIV infection	Total Stroke^a^							Ischemic stroke					Hemorrhage stroke				
	HIV group			Comparison group			HR (95% CI)^c^	HIV group		Comparison group		HR (95% CI)^c^	HIV group		Comparison group		HR (95% CI)^c^
	No.	No. Event	Rate^b^	No.	No. Event	Rate^b^		No. Event	Rate^b^	No. Event	Rate^b^		No. Event	Rate^b^	No. Event	Rate^b^	
**1998–1999**	1302	45	3.21	5208	160	2.44	1.51 (1.08–2.10)	28	2.00	89	1.36	1.73 (1.13–2.65)	11	0.78	47	0.72	1.18 (0.61–2.27)
**2000–2002**	1786	22	1.40	7144	155	2.21	0.75 (0.48–1.18)	11	0.70	87	1.24	0.68 (0.39–1.28)	8	0.51	42	0.60	0.95 (0.45–2.04)
**2003–2005**	2873	36	1.91	11492	110	1.39	1.44 (0.98–2.11)	20	1.06	69	0.87	1.33 (0.80–2.20)	10	0.53	27	0.34	1.52 (0.72–3.18)

Abbreviations: No., Number CI, confidence interval; HIV, human immunodeficiency virus; HR, hazard ratio.

^a^Patients with undetermined stroke were excluded in the analysis by stroke subtypes (n = 79).

^b^Incidence rate per 1000 person-years.

^c^Adjusted for age, sex, and comorbidities including diabetes, hypertension, hyperlipidemia, chronic kidney disease, cancer, coronary heart disease and atrial fibrillation.

P values of test for interaction effect between HIV infection and year of diagnosis: 0.034 for total stroke, 0.056 for ischemic stroke and 0.643 for hemorrhage stroke.

### HIV infection and the risk of stroke by follow-up period

**Fig 2:**
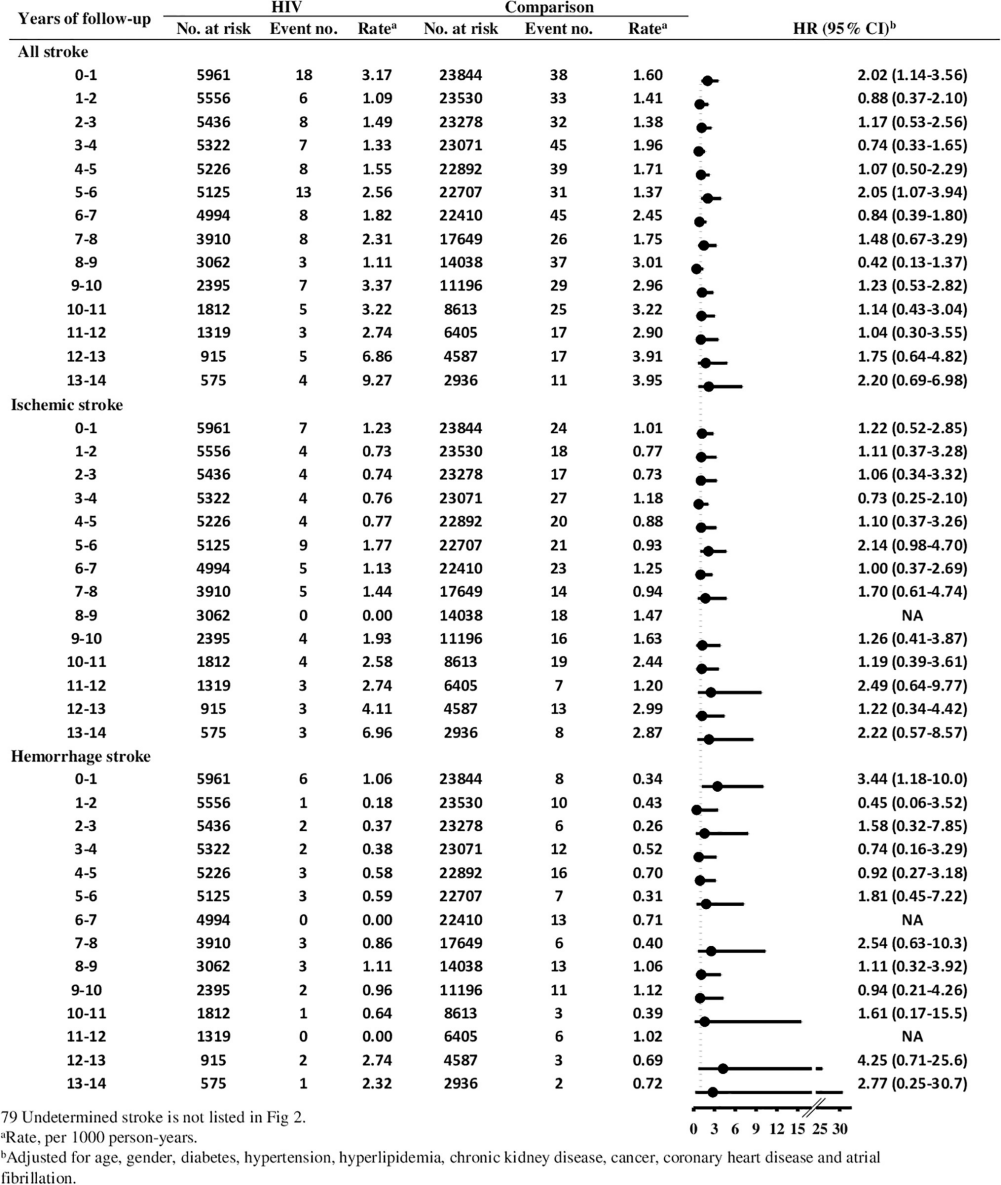
Risk of stroke in association with HIV infection according to time of follow-up.

Fig 2. shows the analyses of the association between HIV infection and the risk of developing stroke stratified by 1-year intervals of follow-up duration. The adjusted HRs were slightly increased in the first year and in years 5–6 after HIV diagnosis than in other time periods, but the difference in risk was not statistically significant (p = 0.94). The HRs for ischemic stroke and hemorrhagic stroke also did not differ over follow-up time (p = 0.38 and p = 0.90, respectively).

The models were adjusted for age, sex, and comorbidities including diabetes, hypertension, hyperlipidemia, chronic kidney disease, cancer, coronary heart disease, and atrial fibrillation. Patients with undetermined stroke were excluded in the analysis by stroke subtypes (n = 79). HR indicates hazard ratio; CI, confidence interval.

## Discussion

The investigation of HIV infection as an independent risk factor of stroke incidence in different countries is worth noting. Our findings provide information for Asian subjects that individuals with HIV infection among a younger age cohort had increased risk of overall and ischemic stroke, and HIV infection-related risk of stroke was higher among subjects diagnosed in the early cART era.

Epidemiological studies in different areas and with different ethnic groups revealed variations in the incidence of stroke among people with HIV infection. In general, studies from Asia yielded lower incidence. Chow et al. reported that the incidence rate of ischemic stroke per 1000 person-years was 5.27 in the HIV cohort and 3.75 in patients without HIV [8], and the incidence of hemorrhage stroke per 1000 person-years was 2.29 in the HIV-infected group compared with 1.23 in non-HIV group [12] in a local US health care system. One recent study in Taiwan showed the incidence rates of total stroke, ischemic stroke, and hemorrhagic stroke were 2.53 vs. 1.4; 1.87 vs. 1.01; 0.66 vs. 0.39 per 1000 person-years in the HIV cohort compared with the non-HIV cohort [14]. Another recent study found 2.2 cardiovascular events per 1000 person-years in the TREAT Asia HIV Observational Database [25]. Our observations revealed that the incidence rate of overall stroke per 1000 person-years was 2.12 among patients with HIV infection and 1.98 among the comparison group, which is lower than the studies reported in Western countries [5, 7–9, 11, 12], but similar to those in Asia [14, 25]. The reasons for the lower incidence rate of stroke in HIV-infected individuals in Asia remain unclear. Explanations may include the differences in study methods such as data sources, survey methods, time periods, the inclusion criteria for subject selection, and different population genetics.

The strength of the associations in our analyses, which showed an adjusted HR of 1.21 (95% CI 0.98–1.51) for total stroke and 1.23 [95% CI 0.93–1.64]) for ischemic stroke, was weaker than that observed in a Danish study [5] and a recent study in Taiwan [14] (HR [95% CI] for total stroke, 1.60 [1.30–1.95] and 1.83 [1.58–2.13], respectively), but similar to findings in a US study (HR [95% CI], 1.21 [1.01–1.46] for ischemic stroke) [8]. The statistical non-significance may reflect insufficient statistical power to detect the moderate association because of the relatively lower incidence of stroke and the smaller number of outcome events in our analysis than in the US study [8]. Furthermore, our study suggests variability of the association across patient subgroups of age and calendar year of diagnosis of HIV infection, which warrants further investigation.

Few studies have reported the association between HIV infection and risk of stroke by age and sex. Our observation was in line with the finding of Chow et al. [8], which showed a greater HIV infection-related risk of ischemic stroke in young people and women. However, their study did not report the results of testing for interaction [8] with age and sex. We found that the difference in the risk of total stroke and ischemic stroke associated with HIV infection was statistically significant by age but not by sex. Previous studies have also indicated that the risk of hemorrhagic stroke associated with HIV infection increased particularly in younger patients and with more advanced disease [11, 12, 14, 26]. However, it may not be appropriate to make conclusions about the risk of hemorrhagic stroke in our analysis because the number of events was very small in the age and sex stratifications.

The potential mechanisms of ischemic stroke and hemorrhagic stroke in patients with HIV infection are multifactorial. HIV-related causes of ischemic stroke include aneurismal formation, vasculitis, accelerated atherosclerosis, HIV-associated cerebral blood vessel disease, opportunistic infection or neoplasia, cardioembolism, coagulopathy, and HIV-associated hyperviscosity [1, 27]. Possible HIV-related causes of hemorrhagic stroke include HIV-associated aneurysmal vasculopathy [28], vasculitis [29], immune thrombocytopenia [30], AIDS associated tumors, or infection [31, 32].

Our observations showed that the mean age diagnosed with stroke was 7-years younger in the HIV infection cohort than in the comparison group. This result is consistent with previous studies, which reported that patients with HIV developed stroke younger than those without HIV infection in individuals without traditional risk factors [33], even with good immune function [34], both in ischemic stroke [8, 33] and in hemorrhagic stroke [12, 26]. In Africa, patients with HIV that developed stroke were younger with a median age of 33.4 years in South Africa [33] and 39.8 years in Malawi [35]. Stroke incidence is usually low in young individuals and rises exponentially with age [36], because the vascular risk factors of stroke do not occur frequently in young individuals [37]. The remarkably high risk of stroke association with HIV infection in young people, and the younger age at diagnosis of stroke in the HIV infection group than in the comparison group implies that HIV infection plays an important role in young stroke.

Our finding in the analysis stratified by calendar year of diagnosis is consistent with the study by Alvaro-Meca et al., which revealed a decline in the stroke risk among HIV-infected individuals in more recent years after the introduction of HAART (highly active antiretroviral therapy) [21]. There may be a number of reasons for this decline. First, the adverse side effects of older antiretroviral regimens or more effective treatments in the recent era has led to seeing a higher stroke risk in the early HAART epoch than in more recent periods. Second, limited tools for making a stroke diagnosis in the earlier period might have resulted in an overestimated misdiagnosis [38, 39]. For example, HIV encephalopathy [40] or an HIV-related CNS opportunistic infection can mimic stroke [41].

In a stratified analysis by follow-up duration, we observed greater HRs for stroke in the first year after the diagnosis of HIV infection, but the interaction effect with follow-up time was not statistically significant. However, the number of stroke events in the HIV infection group in the stratifications of follow-up time was small. Further studies with larger sample sizes may help clarify this issue. From a prevention point of view, the importance of earlier risk factor correction and stroke prevention should still be emphasized once the definite diagnoses of HIV infection are established.

Our study has some limitations. First, patient characteristics including smoking history and status, BMI, and laboratory data, are not available in the claims data. Therefore, we were unable to assess the extent to which the confounding effects by these factors, if they exist, could explain the observed association between HIV infection and the incidence of stroke. However, such a confounding effect is unlikely to fully account for all the observed associations, particularly for the subgroups of young patients and those diagnosed in earlier years, in which the associations were relatively strong. Further studies that collect these variables could help clarify this issue. Second, our necessary reliance on administrative claims data recorded by physicians and hospitals to establish diagnoses of HIV infection and stroke are less accurate than those designed in prospective settings. To minimize bias and strengthen the reliability, only patients with Catastrophic Illness Certificates of HIV infection were included. Issuing the certificates requires approval by an expert committee after review of the medical records. Stroke events were defined as hospitalized patients whose first major diagnoses were stroke. Third, information on the treatments of HIV infection is not available in this study. Thus, we were unable to evaluate the association between the treatments and the stroke risk in patients with HIV infection in this post-cART era. Fourth, our study based on observational claims data analysis cannot establish the mechanism of developing stroke in association with HIV infection. Finally, the non-significant results may be due to insufficient statistical power to detect the moderate association or the small number of events in the analyses of hemorrhagic stroke.

## Conclusions

This nationwide population-based study in Taiwan reveals that young individuals with HIV infection have an elevated risk of subsequent stroke. Ischemic stroke risk was higher in the early stage of antiretroviral therapy and declined in the more recent era. An etiology survey and risk factor control for stroke prevention should be provided to young HIV-infected individuals aggressively and as early as possible.

## Supporting information

S1 Table The disease and ICD-9-CM code. (DOCX)

## Abbreviations

AIDS: acquired immune deficiency syndrome
c ART: combination antiretroviral therapy
CI: confidence interval
CIC: catastrophic illness certificate
CVD: cardiovascular disease
HAART: highly active antiretroviral therapy
HIV: Human immunodeficiency virus
ICD: International Classification of Diseases
ICD-9-CM: International Classification of Diseases, Ninth Revision, Clinical Modification
LHID: Longitudinal Health Insurance Database for one million people insured
NHI: National Health Insurance
NHIRD: National Health Insurance Research Database
PLWHA: people living with HIV infection
SIR: standardized incidence ratio
