# Supplementary material for: Incidence of stroke in patients with HIV infection: A population-based study in Taiwan
Source: PLoS One. 2019 May 22;14(5):e0217147. doi: 10.1371/journal.pone.0217147 (PMC6530842; doi:10.1371/journal.pone.0217147)
Supplement: S1 Table — (DOCX) [file pone.0217147.s001.docx]

| **S1 Table. The disease and ICD-9-CM code.** | |
| --- | --- |
| **Disease** | **ICD-9-CM code** |
| HIV disease | 042 |
| Asymptomatic HIV infection status | V08 |
| Stroke | 430-438 |
| Hemorrhage stroke | 430-432 |
| Ischemic stroke | 433, 434, and 435.9 |
| Undetermined stroke | 435-438 exclude 435.9 |
| Diabetes Mellitus | 250 |
| Chronic kidney disease (CKD) | 580-587 |
| Hypertension | 401-405 |
| Coronary heart disease (CHD) | 410-414 |
| Cancer | 140-208 |
| Dyslipidemia | 272 |
| Atrial fibrillation (AF) | 427.31 |
